# Mimicking hypersensitivity pneumonitis as an uncommon initial presentation of chronic granulomatous disease in children

**DOI:** 10.1186/s13023-017-0719-9

**Published:** 2017-10-26

**Authors:** Hui Liu, Jinrong Liu, Huimin Li, Yun Peng, Shunying Zhao

**Affiliations:** 1 Department of Respiratory Medicine, Beijing Children’s Hospital, Capital Medical University, National Center for Children’s Health, Nanlishi Road 56, Xicheng District, Beijing, China; 2 Imaging Center, Beijing Children’s Hospital, Capital Medical University, National Center for Children’s Health, Nanlishi Road 56, Xicheng District, Beijing, China

**Keywords:** Chronic granulomatous disease, Hypersensitivity pneumonitis, *A. fumigatus*, Glucocorticoid, Children

## Abstract

Dry cough, dyspenea and diffuse centrilobular nodules in both lungs of radiologic findings similar to hypersensitivity pneumonitis (HP) are rare initial presentation in chronic granulomatous disease (CGD). CGD is remarkable for increased susceptibility to bacterial and fungal infections as well as high sensitivity to inciting antigens such as *Aspergillus* species due to dysregulated inflammation. We identified three children who had an initial presentation mimicking HP and were subsequently diagnosed as CGD. All patients developed invasive pulmonary *A. fumigatus* infection (IPAI) following systemic glucocorticoid therapy. Two of the three patients were found to have mutations in *NCF1* gene and one patient in *NCF2* gene. As HP is uncommon in children, we should consider the possibility of CGD in children with HP, even in mimicking HP patients with suggestive inhalation history and negative fungal cultures. A prompt diagnosis of CGD is essential to enable initiation of prophylactic antibacterial and antifungal therapies.

## Letter to the editor

Chronic granulomatous disease (CGD) is characterized by recurrent and severe bacterial and fungal infections as well as excessive inflammation, which are most prominent in gastrointestinal and genitourinary tracts, such as granulomata mimicking Crohn’s disease [1].

An exuberant respiratory inflammation induced by the exposure to inciting antigens and clinically manifested as hypersensitivity pneumonitis (HP) or allergic bronchopulmonary aspergillosis (ABPA) has also been described in CGD [2–7]. However, HP as an initial presentation of CGD is uncommon and has never been reported. Here we review three children who had an initial presentation mimicking HP, developed invasive pulmonary *A. fumigatus* infection (IPAI) following systemic glucocorticoid therapy and were subsequently diagnosed as CGD.

## Case presentation

### Case 1

A 4-year-old boy was admitted to the hospital on September 8, 2011 after 3 weeks of dry cough, progressive dyspnea and fever. He lived in a fruit stall with many rotten fruits inside. He had a history of pneumonia at 3 months old. He also had a history of severe eczema and seasonal rhinitis at one year old.

**Fig. 1:**
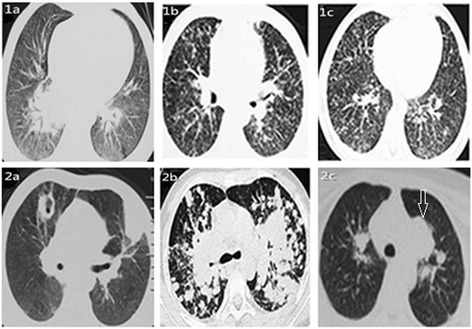
Chest HRCT scans showing the presence of diffuse nodular opacities and slight ground-glass in bilateral inferior fields (1a; on admission) and consolidation in left upper lobe and cavity in right upper lobe (2a; after treatment for three weeks) in case 1; bilaterally diffuse ill-defined centrilobular nodules and slight ground-glass (1b; on admission) and multi-nodules fused into pieces more in upper lung (2b; after treatment for 3 weeks) in case 2; and bilaterally diffuse ill-defined centrilobular nodules and slight ground-glass (1c; on admission), consolidation with halo (arrow) in left upper lobe (2c; after treatment for one month) in case 3

**Fig. 2:**
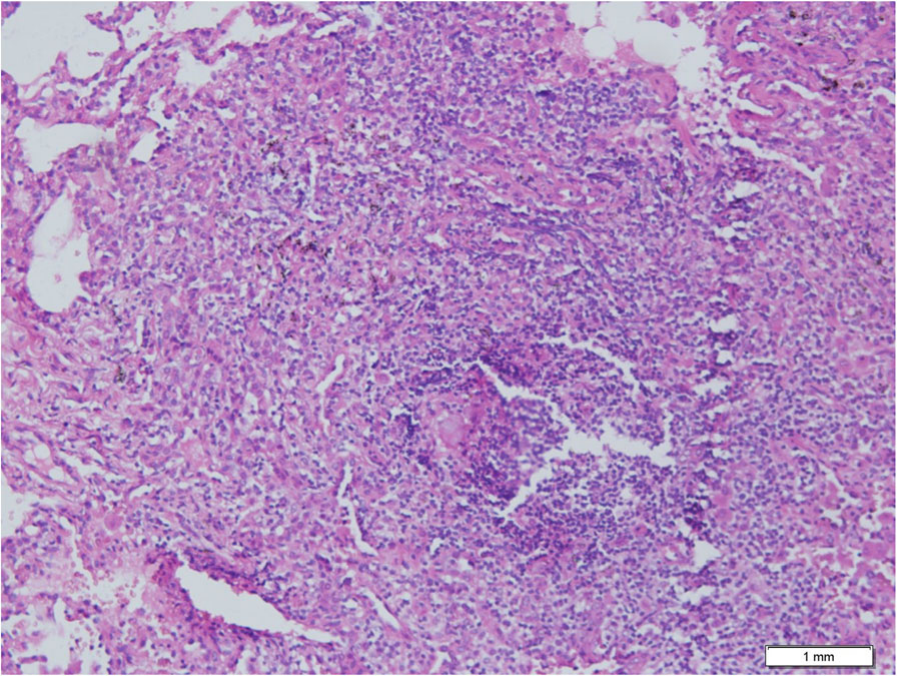
Pathological findings of lung biopsy (original ×200) showing bronchiolo centric lymphocytic infiltrates and non-necrotizing granulomas in lung tissue (case 1)

On admission, his oxygen saturation at rest was 92%, and decreased to 86% after walking. Bilateral basilar rales were noted on auscultation. Chest high-resolution CT (HRCT) scan showed diffuse nodular opacities and slight ground-glass (Fig. 1a). Cultures revealed no evidences of mycobacteria, fungi and viruses. A specimen taken from video-assisted lung biopsy of the right lower lobe revealed bronchiolo centric lymphocytic, and non-necrotizing granulomas and no evidence of fungal or bacterial elements (Fig. 2). Bronchoalveolar lavage fluid (BALF) from his right middle lobe consisted of 46% macrophages, 2% eosinophils and 52% T cells, with a CD4^+^/CD8^+^ ratio of 0.62. Fungal and mycobacterial cultures of BALF were negative, as were T-cell interferon-γ release assays for tuberculosis and polymerase chain reactions for pneumocystis jirovecii. After exclusion of infectious agents, diagnosis of mimicking HP probably due to inhalation of rotten fruit elements was made. Treatment with 1 mg/kg/day prednisone was initiated and clinical symptoms improved after 3 days.

Three weeks after the beginning of tapered prednisone, he developed fever and cough with purulent sputum. HRCT found consolidation in left upper lobe and cavity in right upper lobe (Fig. 2a). Sputum culture was positive for *A. fumigatus* three times. Parenteral voriconazole therapy for 2 months followed by oral voriconazole was administered for 6 months until lung lesions disappeared completely. In consideration of the patient’s progressive course, he was referred to immunological test. Dihydrorhodamine-1,2,3 (DHR) test showed the absence of neutrophil oxidative burst consistent with CGD. Gene mutation analyses revealed compound heterozygous mutations (c.278A > T and c.475delA) in *NCF2* gene, indicating autosomal recessive CGD [8]. Continuous prophylactic treatment with trimethoprim-sulfamethoxazole and itraconazole were administered, and no infection recurred in a follow-up period of 4 years.

### Case 2

An 8-year-old girl was admitted to the hospital on February 15, 2015 because of high spiking fever and chills, dry cough, progressive dyspnea and chest stuffy for 20 days. Twenty-four days ago she had burned decayed cornhusks with her brother (case 3) for 4 h. She had a history of severe eczema and seasonal rhinitis at 3 years old.

On admission, her oxygen saturation at rest was 93%, and decreased to 84% after walking. Bilateral basilar rales were noted on auscultation. HRCT scan showed the presence of bilaterally diffuse centrilobular nodules and slight ground-glass (Fig. 1b). BALF from her right middle lobe consisted of 41% macrophages, 3% eosinophils and 56% T cells, with a CD4^+^/CD8^+^ ratio of 0.65. Fungal and mycobacterial cultures of BALF were negative. The diagnosis of mimicking HP was made. A treatment of 1 mg/kg/day prednisone was initiated, and clinical symptoms improved after 2 days.

As prednisone being tapered after 3 weeks, she developed fever, racking cough with purulent sputum and chest stuffy. Repeated HRCT showed multi-nodules bilaterally distributed along bronchi and part of multi-nodule fused into pieces more in upper lung (Fig. 2b). Specific IgG antibody to *A. fumigatus* was positive. Both culture of sputum and BALF from right middle lobe found *A. fumigatus.* Amphotericin liposome B was taken for 2 months followed by oral voriconazole for one year. The nodes disappeared and the pieces shrank. In consideration of the fact that his brother had a history of perianal abscess and was diagnosed as CGD by means of an abnormal DHR test, she was referred to DHR test, which was consistent with CGD. Sequencing of genomic DNA revealed homozygous mutation (c.541delG) in *NCF1* gene, indicating autosomal recessive CGD [9]. Continuous prophylactic antibiotic treatment was administered, and no symptoms recurred anymore.

### Case 3

A 5-year-old boy with high spiking fever and chills, dry cough and progressive dyspnea was admitted to the hospital at the same time with his sister (case 2). He had the same exposure to decayed cornhusks. He had a history of perianal abscess and severe eczema at 3 months old, and no history of previous pneumonia, asthma, or exercise intolerance.

On admission, his oxygen saturation at rest was 95%, and decreased to 87% after walking. Bilateral basilar rales were noted on auscultation. HRCT revealed bilaterally diffuse ill-defined centrilobular nodules and slight ground-glass (Fig. 1c). Lymphocytosis of 50% with a CD4^+^/CD8^+^ ratio of 0.73, 48% macrophages and 2% neutrophils were shown in BALF. Fungal and mycobacterial cultures for BALF were negative. The diagnosis of mimicking HP was made. Treatment with 1 mg/kg/day oral prednisone was initiated and clinical symptoms improved after 3 days. A slow taper of prednisone was continued after 3 weeks.

Despite the lack of aggravated symptoms, specific IgG antibody to *A. fumigatus* was positive and his chest HRCT revealed a nodular consolidation with halo sign in left upper lobe (Fig. 2c). Oral voriconazole was taken for 4 months. The halo disappeared and the consolidation shrank. In consideration of his history of perianal abscess, he was referred to DHR test, and the result was consistent with CGD. He was subsequently confirmed to have the same *NCF1* gene mutation as his sister. Prophylactic antibiotic treatment was continuously administered. No extra symptoms occurred anymore.

The clinical data relating to HP and IPAI of the 3 cases are summarized in Table 1. Antecedent history and genetic tests for CGD are summarized in Table 2.

**Table 1:** Clinical, physiological, radiographical, and pathological data relating to mimicking HP and IPAI in the 3 CGD patients

CGD patient	Case 1	Case 2	Case 3
Age (yr)	4	8	5
Sex	male	female	male
Data of mimicking HP			
Exposure to an offending environment	Antigens probably relating to rotten fruits	Antigens probably in musty cornhusks	Antigens probably in musty cornhusks
Symptom/sign	Dry cough, dyspnea, fever, bilateral basilar rales	Spiking fever with chills, dry cough, dyspnea, chest stuffy, bilateral basilar rales	Spiking fever with chills, dry cough, dyspnea, bilateral basilar rales
Pulmonary function	FEV1, 0.48 L (51.2% predicted); FVC, 0.65 L (68.3% predicted)	FEV1, 0.8 L (58.7% predicted); FVC, 0.92 L (58.9% predicted); DLCO, 6.46 ml/min/mmHg (46.6% predicted)	FEV1, 0.59 L (57.3% predicted); FVC, 0.72 L (70.5% predicted)
Chest HRCT scan	Diffuse nodular opacities and slight ground-glass in bilateral inferior field	Bilaterally diffuse ill-defined centrilobular nodules and slight ground-glass	Bilaterally diffuse ill-defined centrilobular nodules and slight ground-glass
BALF cells	AM: 46%; Lym: 52%; Eos: 2%; CD4^+^/CD8^+^: 0.62	AM: 41%; Lym: 56%; Eos: 3%; CD4^+^/CD8^+^: 0.65	AM: 48%; Lym: 50%; Neu: 2%; CD4^+^/CD8^+^: 0.73
Lung biopsy	Bronchiolo centric lymphocytic, non-necrotizing granulomas and no evidence of fungal or bacterial elements	Not available	Not available
Bacterial/viral/fungal cultures	Negative	Negative	Negative
Treatment	1 mg/kg/d prednisone	1 mg/kg/d prednisone	1 mg/kg/d prednisone
Data of IPAI			
HRCT scan finding	Consolidation in left upper lobe and cavity in right upper lobe	Multi-nodules bilaterally distributed along bronchi and part of multi-nodules fused into pieces more in upper lung	A nodular consolidation with halo sign in left upper lobe
Bacterial/viral/fungal cultures	*A. fumigatus*	*A. fumigatus*	*A. fumigatus*
Treatment	Infusion of voriconazole for 2 months followed by oral voriconazole for 6 months	Infusion of amphotericin liposome B for 2 months followed by oral voriconazole for one year	Oral voriconazole for 4 months

*HP* hypersensitivity pneumonitis, *IPAI* invasive pulmonary A.fumigatus infection, *HRCT* high-resolution computer tomography, *BALF* bronchoalveolar lavage fluid, *AM* Alveolar macrophages, *Lym* lymphocytes, *Neu* neutrophils, *Eos* eosinophils, *FEV1* forced expiratory volume in one second, *FVC* forced vital capacity, *DLCO* decreased lung diffusion of carbon monoxide

**Table 2:** Clinical, radiographical, laboratory and genetic data for CGD of the 3 patients

CGD patient	Case 1	Case 2	Case 3
Antecedent history	Pneumonia at 3 months old, eczema and seasonal rhinitis at one year old	Eczema and seasonal rhinitis at 3 years old	Perianal abscesses and eczema at 3 months old
Igs	IgG 12.2 g/L, IgM1.72 g/L, IgA2.59 g/L, IgE 598.9 IU/mL	IgG 26.8 g/L, IgM1.12 g/L, IgA4.55 g/L, IgE 3000 IU/mL	IgG 26.6 g/L, IgM1.29 g/L, IgA4.23 g/L, IgE 365.9 IU/mL
Lymphocyte subsets in peripheral blood	NK cells (9.3%), B cell (11.9%); CD4^+^ cells (43.2%), CD8^+^ cells (32.85%)	NK cells (4%), B cell (14%); CD4^+^ cells (39%), CD8^+^ cells (37%)	NK cells (11%), B cell (17%); CD4^+^ cells (33%), CD8^+^ cells (36%)
DHR test	No uptake in neutrophil oxidative burst after phorbolmyristate acetate stimulation	No uptake in neutrophil oxidative burst after phorbolmyristate acetate stimulation	No uptake in neutrophil oxidative burst after phorbolmyristate acetate stimulation
Gene mutation	*NCF2* gene (compound heterozygosity mutation c.278 A > T and c.475delA)	*NCF1* gene (homozygous mutation c.541delG)	*NCF1* gene (homozygous mutation c.541delG)

*CGD* chronic granulomatous disease, *DHR* dihydrorhodamine-1,2,3, *Igs* immunoglobulins, *NK* natural killer

## Discussion

In CGD patients, hypersensitivity to *Aspergillus* clinically manifested as HP and ABPA occurs after exposure to a variety of antigens, and is characterized by the constitutional symptoms and non-necrotizing granulomas in walls of alveoli and airways. However, HP as the first manifestation of CGD is rare in children.

Our cases illustrate that they had the clinical presentations similar to HP, including the history of exposure to potential inciting antigens, lymphocytosis in BALF, compatible image features, poorly formed granulomas on lung biopsy in one patient (case 1), and a favorable response to systemic glucocorticoids. However, these patients did not fully meet the diagnostic criteria for HP published by Venkatesh and Wild [10].

Lung biopsy in one patient (case 1) revealed non-necrotizing granulomatous pneumonitis with variable airspace organization. It is necessary to rule out the pulmonary granulomatous process related to the underlying CGD. Granulomas in patients with CGD are typically small, containing central neutrophil micro-abcesses surrounded by epithelioid histiocytes and giant cells and spreading in airways or lung parenchyma [11, 12]. Unlike CGD, the granulomas of our patient were loosely formed and relatively larger, spread through airway to lymph tract, and did not contain central micro-abcesses, similar to the adult CGD patient reported by Katsuya and colleagues [5].

Recently, Esenboga et al. reported that a 16-year-old patient atypically presented with chronic HP caused by close contact with pigeons and exposure to their allergens before the diagnosis of CGD caused by homozygous deletion mutation in *NCF1* [13]. Like the present cases, the patient recovered by allergen avoidance combining with the use of anti-inflammatory drug. Meanwhile in another study that followed up 33 patients with X-linked CGD for 10 years, four patients developed interstitial lung disease (ILD). Two of the four patients, one 20 years old and the other 23 years old, were diagnosed with HP through CT images and histopathological examination, probably caused by breathing in dust in a plant and an unknown antigen, respectively [14]. However, the clinical symptoms in the two patients were mitigated only by allergen avoidance, suggesting that CGD patients are more susceptible to hyperinflammation resulting from inhalation of antigens.

The combination of HP/ABPA features has been described in 2 CGD patients with a gradually progressive course over many years [6, 7]. Interestingly, case 2 had the ABPA features of elevated total serum IgE (3000 IU/mL). However, in ABPA patients, acute course or exacerbation may present nodular pulmonary infiltrates, centrilobular nodules and bronchiectasis on HRCT [15]. Lymphocytosis in BALF is uncommon in ABPA.

The mechanisms underlying this hyperinflammation are still under investigation. A plausible explanation is that the reduced ROS cannot adequately inhibit the production of inflammatory cytokines [16], and that this ROS deficit in CGD allows for the continuous production of inflammatory cytokines, resulting in immune dysregulation or hyperinflammation. Thus, one of the effective therapeutic approaches for such hyperinflammation is the use of corticosteroids or immunosuppressive drugs [17]. Indeed, infliximab, a chimeric antibody against tumor necrosis factor-α (TNFα), has shown therapeutic efficacy for refractory CGD colitis, as TNFα is thought to play a critical role in granuloma formation in CGD.

Although the symptoms of HP were improved rapidly after systemic corticosteroid treatment, the present patients suffered worsening disease after 3 weeks’ treatment, and the clinical, radiological and positive *A. fumigatus* culture findings are consistent with the diagnosis of IPAI, which is a major life-threatening infection among CGD patients [18]. **There are several reasons for the occurrence of IPAI. Firstly, patients with CGD are prone to develop characteristic invasive fungal infections due to** ***Aspergillus*** **specie. Secondly, prolonged steroids therapy makes a contribution to occurrence of IPAI by increasing the patient’s susceptibility to infection. Thirdly, the risk of** ***Aspergillus*** **infections in the present patients is higher than that of other pathogens infections because of the exposure to rotten fruits or decayed cornhusks which are considered as** ***Aspergillus*** **spores-rich environment. The occurrence of IPAI makes it easier to diagnose CGD, but which would be detected much earlier in the inflammatory period.**

There are two previous reports of 10 patients who developed fulminant mulch pneumonitis (FMP) as emergency presentation of CGD [19, 20]. Unlike our series, the 10 patients presented acute, rapid, often fatal, invasive aspergillosis symptoms after an identifiable exposure to organic material; culture results were positive for *Aspergillus* before systemic treatment of glucocorticoids, and 5 of the 10 patients died despite the treatment of antifungals and steroid. **Based on the disease duration of the present patients, similar inflammatory responses characterized by lung granuloma formation, lymphocytosis in BALF and** ***Aspergillus*** **specific IgG antibodies could be viewed as the subacute end of the acute FMP spectrum caused by inhalation of variable doses of** ***Aspergillus*** **spores.**

The incidence of HP is probably much higher in CGD patients, especially in children. CGD should be first excluded by diagnostic DHR testing before steroids therapy in patient with newly diagnosed HP or mimicking HP. Furthermore, it should be noted that the combined application of antifungal drugs and steroids or other anti-inflammatory drugs is more appropriate from the beginning of the treatment in HP patients with suspected CGD, especially in patients with a history of fungal exposure.

## Conclusions

**CGD should be considered in children with HP, even in mimicking HP patients with suggestive inhalation history and negative fungal cultures before steroids therapy.** A prompt diagnosis of CGD is essential to enable initiation of prophylactic antibacterial and antifungal therapies.

## Abbreviations

ABPA: Allergic bronchopulmonary aspergillosis
BALF: Bronchoalveolar lavage fluid
CGD: Chronic granulomatous disease
DHR: Dihydrorhodamine-1,2,3
HP: Hypersensitivity pneumonitis
HRCT: High-resolution computer tomography
IPAI: Invasive pulmonary *A.fumigatus* infection
TNFα: Tumor necrosis factor-α
